# Urinary angiotensinogen as a surrogate marker predicting the antiproteinuric effects of angiotensin receptor blockers in patients with overt proteinuria: a multicenter prospective study

**DOI:** 10.1186/s12882-020-01825-6

**Published:** 2020-05-01

**Authors:** Junseok Jeon, Do Hee Kim, Hye Ryoun Jang, Jung Eun Lee, Wooseong Huh, Hye-Young Kim, Dae Joong Kim, Yoon-Goo Kim

**Affiliations:** 1 Division of Nephrology, Department of Medicine, Samsung Medical Center, Sungkyunkwan University School of Medicine, 81 Irwon-ro, Gangnam-gu, Seoul, 06351 Republic of Korea; 2 Division of Nephrology, Department of Medicine, Chungbuk National University Hospital, Cheongju, 28644 Republic of Korea

**Keywords:** Urinary angiotensinogen, Urinary renin, Intrarenal renin angiotensin system, Angiotensin receptor blocker, Proteinuria

## Abstract

**Background:**

Although urinary angiotensinogen (AGT) and renin reflect intrarenal renin-angiotensin system activity and are enhanced in proteinuric chronic kidney disease, the clinical value of urinary AGT and renin levels during antiproteinuric treatment has yet to be determined. We investigated the clinical usefulness of initial urinary AGT or renin to determine the antiproteinuric effects of angiotensin receptor blockers (ARBs).

**Methods:**

This multicenter, prospective, single-arm study included 205 patients with overt proteinuria (urinary protein/creatinine ratio [uPCR] ≥ 1 mg/mg) enrolled between April 2009 and December 2011. All patients were treated with valsartan. The urinary AGT/creatinine ratio (uAGT/Cr) was measured at the baseline and 24 weeks, and the renin/creatinine ratio (uR/Cr) was measured at the baseline. Fifty-six patients were followed-up for 5 years.

**Results:**

The mean age was 47.6 years and 51.2% were male. The mean uPCR was 2.32 mg/mg and the mean eGFR was 63.2 mL/min/1.73m^2^. Natural logarithms (ln) (uAGT/Cr), ln(uR/Cr), and diabetes mellitus were associated with proteinuria decrement (decrease in uPCR ≥1 mg/mg). Ln(uAGT/Cr) was an independent predictor for proteinuria decrement (OR 1.372, 95% CI, 1.068–1.762, *P* = 0.013). Among the 56 patients followed-up for 5 years, Δln(uAGT/Cr) at 24 weeks was an independent predictor for uPCR < 1 mg/mg at 5 years (OR 0.379, 95% CI, 0.20–0.715, *P* = 0.003).

**Conclusions:**

Our study demonstrates the potential role of both baseline urinary AGT and changes in urinary AGT during the initial 24 weeks as surrogate markers predicting the antiproteinuric effects of ARBs in patients with overt proteinuria.

## Background

Inhibition of the renin-angiotensin system (RAS) is a cornerstone of managing chronic kidney disease (CKD) patients with proteinuria including diabetic nephropathy and glomerulonephritis. Previous studies have shown that angiotensin-converting enzyme inhibitors (ACEi) or angiotensin II receptor blockers (ARBs) that inhibit RAS mitigate the progression of CKD by reducing proteinuria beyond their blood pressure-lowering effects [1, 2]. Regulation of local RAS is independent of systemic RAS in various tissues, including the kidneys, and intrarenal RAS has all RAS components in contrast to other organs [3]. Activated intrarenal RAS plays an important role in the pathogenesis of hypertension and CKD progression [4, 5]. Urinary angiotensinogen (AGT) and renin reflect the activity of the intrarenal RAS [6–8]. Since the enzyme-linked immunosorbent assay (ELISA) system measuring AGT was developed, several clinical studies have demonstrated that urinary AGT excretion increases in patients with hypertension [9], CKD [7, 10], or diabetes mellitus (DM) [11] regardless of plasma AGT levels.

The degree of proteinuria is strongly associated with CKD progression and cardiovascular disease [12]. Prediction or enhancement of the antiproteinuric effects of RAS inhibitors are important issues in the management of CKD [13, 14]. Previous studies have reported that urinary AGT excretion is positively correlated with the degree of proteinuria [7, 15] and is decreased by RAS inhibitors [9, 16]. Increased urinary AGT excretion may proceed the aggravation of albuminuria [11, 17]. On the other hand, urinary renin excretion may also be a potential marker for intrarenal RAS activity or a predictor of the antiproteinuric effects of RAS inhibitors [8, 18]. However, few studies have investigated the relationship between the degree of urinary AGT or renin excretion and the antiproteinuric effects of RAS inhibitors [8, 16].

In this prospective study, we aimed to investigate the clinical relevance of urinary AGT or renin in the treatment of proteinuria by analyzing the association between urinary AGT or renin excretion and the antiproteinuric effects of RAS inhibitors.

## Methods

### Study design and patient selection

This multicenter, prospective, single-arm study was conducted at eleven tertiary hospitals in South Korea. A total of 323 adult patients under the age of 70 with overt proteinuria (urinary protein to creatinine ratio [uPCR] ≥ 1 mg/mg) were enrolled among patients who visited the nephrology clinic between April 2009 and December 2011. Blood pressure was relatively well controlled, ranging from 100/60 to 160/100 mmHg, in all patients. Low-salt diet was thoroughly educated in all patients at the time of enrollment. The exclusion criteria were as follows: an estimated glomerular filtration rate (eGFR) less than 30 mL/min/1.73 m^2^, the use of non-interruptible drugs that affect proteinuria, patients who received immunosuppressive treatment during the preceding 6 months, severe proteinuria with uPCR > 10 mg/mg, serum albumin < 2.5 g/dL or refractory edema, uncontrolled DM with HbA1C > 9.0%, underlying conditions that may affect intrarenal RAS activity (e.g., renal artery stenosis, primary aldosteronism, or pheochromocytoma), cardiovascular events during the preceding 12 months (e.g., myocardial infarction, unstable angina, coronary artery bypass surgery, percutaneous transluminal coronary angioplasty, cerebrovascular accident, or transient ischemic attack), hypokalemia or hyperkalemia (K ≤ 3.5 or ≥ 5.5 mmol/L, respectively), abnormal liver function (aspartate transaminase or alanine transaminase higher than twice the upper limit of the normal range), allergic reaction to angiotensin-converting enzyme inhibitors (ACEi) or angiotensin II receptor blockers (ARBs), or pregnant or nursing patients. We enrolled patients with uPCR ≥1 mg/mg and < 10 mg/mg to maximize the clinical effect of antiproteinuric effect and exclude full-blown nephrotic syndrome patients who are at high risk for rapid progression with RAS inhibitor treatment. Patient with advanced CKD (eGFR < 30 mL/min/1.73 m^2^) were excluded considering the relative high risk-benefit ratio of ACEi/ARB and potential interference of renal function on urinary AGT or renin.

**Fig. 1:**
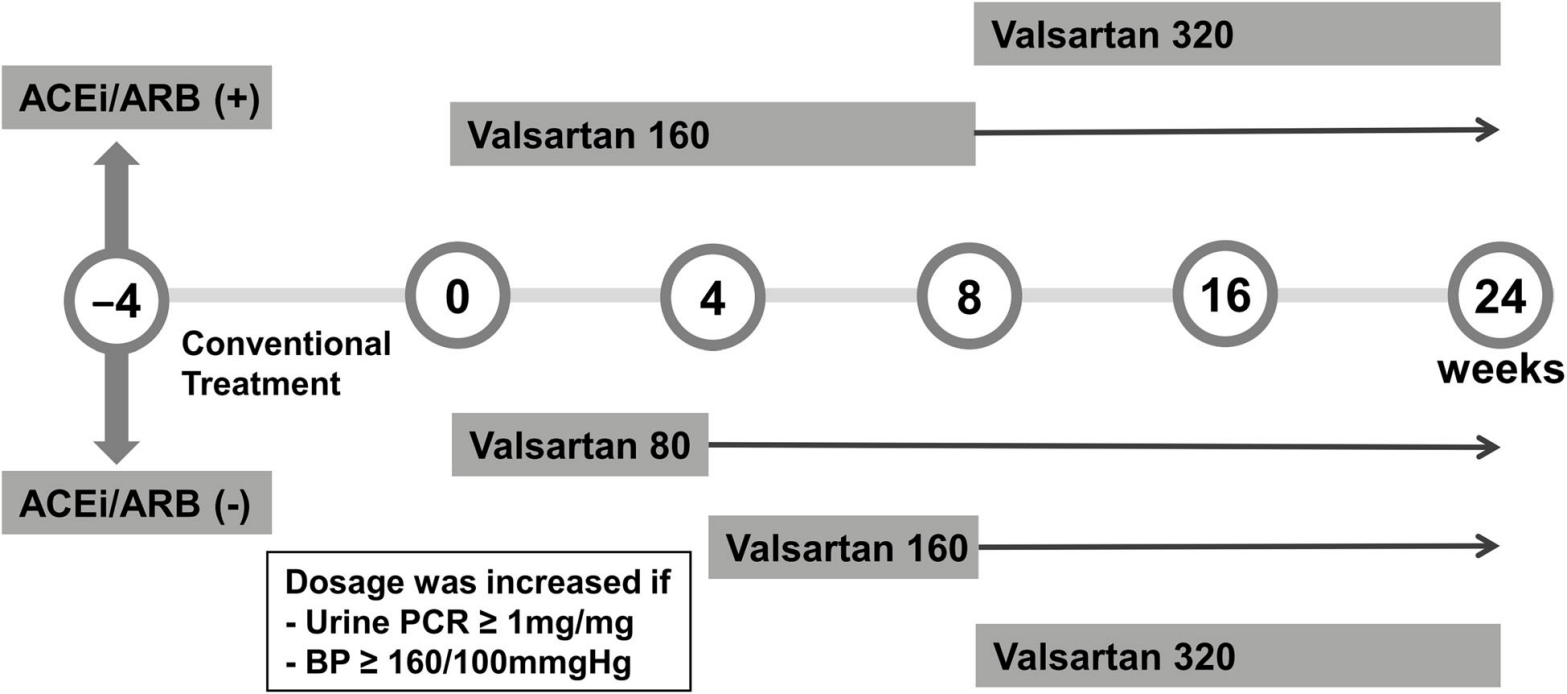
Study design and assessment schedule. A total of 323 patients with overt proteinuria (urinary protein/creatinine ratio [uPCR] ≥ 1 mg/mg) who visited the nephrology clinic at one of 11 tertiary hospitals in Korea between April 2009 and December 2011 was enrolled. Any drugs affecting the activity of the renin-angiotensin system were discontinued. The dosage of valsartan was increased after 8 weeks if the patient presented with a uPCR ≥ 1 mg/mg or blood pressure over 160/100 mmHg. The uPCR and renal function were also measured at 8 weeks and 24 weeks after enrollment for all patients

Study protocol was summarized in Fig. 1. Valsartan was selected based on the extensively studied antiproteinuric effects and the availability of several dose formularies. Patients received only conventional supportive care without immunosuppressive treatment prior to the study period. If patients were already taking ACEi or ARB before the screening, the drugs were discontinued for at least 4 weeks. Valsartan treatment was started at the beginning of the study with 80 mg for patients who had not previously taken ACEi or ARB and 160 mg for those who had been on ACEi or ARB before the enrollment. If uPCR > 1 mg/mg, systolic blood pressure > 160 mmHg, or diastolic pressure > 100 mmHg at 4 weeks or 8 weeks, the dose of valsartan was increased to 160 mg at 4 weeks and then 320 mg at 8 weeks in patients whose initial dose was 80 mg. The dose of valsartan was increased to 320 mg at 8 weeks in patients whose initial dose was 160 mg.

### Sample collection and measurement of intrarenal RAS activity

Clinical data, including age, sex, underlying comorbidities, and blood pressure, were extracted from electronic medical records. Blood pressures were measured at each clinic visit after a rest period in a chair using an automated blood pressure device. Laboratory data, including hemoglobin, albumin, cholesterol, uric acid, blood urea nitrogen (BUN), serum creatinine (sCr), eGFR, electrolytes, and uPCR, were also collected. The eGFR was calculated based on the modified Modification of Diet in Renal Disease using sCr [19]. All urine samples were measured in a central laboratory.

To measure urinary AGT and renin, overnight-fasting early-morning urine samples were collected before starting valsartan treatment. Samples were centrifuged at 2500 rpm for 15 min at 4 °C, and the supernatants were then stored at − 70 °C. The urine samples were thawed and centrifuged at 13,000 rpm for 2 min at 4 °C just prior to use in the enzyme-linked immunosorbent assay (ELISA) for AGT or the radioimmunoassay for renin. Sandwich ELISAs were performed to quantify urinary AGT (uAGT) using mouse monoclonal and rabbit polyclonal antibodies against recombinant human AGT as previously reported [20, 21]. Highly purified human AGT (Calbiochem, Beeston, UK) was used as the standard. The correlation coefficient of the assay was greater than 0.99, and the detection limit of the ELISA system was 0.9 ng/mL. The urinary renin activity was measured using renin radioimmunoassay beads (TFB, Tokyo, Japan) and a Cobra-II gamma counter (Packard Bioscience, Meriden, CT, USA) as previously reported [8]. Prior to measurement, the urine sample was concentrated 5-fold with an Amicon Ultra-10 centrifugal filter device (Millipore, Cork, Ireland). The detection limit of the enzymatic-kinetic assay was 0.10 ng angiotensin I/mL/h. The renin concentration was determined from the angiotensin I-generating activity with 1 ng angiotensin I/mL/h corresponding to 2.6 pg human renin/mL [8, 22].

To correct for the difference in urinary concentrations, the urinary AGT and renin levels were divided by the urinary creatinine level, resulting in a urinary AGT/creatinine ratio (uAGT/Cr) and a urinary renin/creatinine ratio (uR/Cr), respectively. The uAGT/Cr and uR/Cr levels were then compared after natural logarithmic conversion due to skewed distributions. Delta ln(uAGT/Cr) [Δln(uAGT/Cr)] was defined as ln(uAGT/Cr) at 24 weeks minus the baseline ln(uAGT/Cr), and ΔuPCR was defined as uPCR at either 24 weeks or 5 years after enrollment minus the baseline uPCR.

### Follow-up and outcome measures

The uPCR was measured at 8 weeks and 24 weeks after enrollment in all patients (Fig. 1). The concentrations of urine protein were measured by a colorimetric assay. Baseline ln(uAGT/Cr) and ln(uR/Cr), as well as Δln(uAGT/Cr), were compared between patients with an uPCR decrement ≥1 mg/mg (decrement group) and patients with an uPCR decrement < 1 mg/mg (non-decrement group) during valsartan treatment. Furthermore, other possible prognostic factors for proteinuria decrement were analyzed using multivariable logistic regression analysis. Subgroup analyses regarding predictive factors for a uPCR less than 1 mg/mg and changes in eGFR were conducted for 56 patients who were followed up for 5 years after enrollment.

### Statistical analyses

Continuous variables with a normal distribution are expressed as the mean ± standard deviation (SD), and variables with a non-normal distribution are expressed as the median with interquartile range (IQR). Group means were compared using the *t*-test and medians were compared using the Mann–Whitney test. Serial changes were compared using the paired *t*-test or the Wilcoxon signed-rank test, and repeated measures with analysis of variance (ANOVA), as appropriate. Pearson’s correlation analysis was used to test correlations between two continuous variables. Multivariable logistic regression analysis was performed using variables with *P* <  0.1 in the univariable models. For all tests, a *P*-value < 0.05 was considered statistically significant. All statistical analyses were conducted using IBM SPSS software, version 23 (IBM Corporation, Armonk, NY, USA).

## Results

### Baseline characteristics of the study population

After excluding patients who withdrew consent, were lost follow-up, or did not have data for urinary protein/creatinine ratio at 24 weeks of valsartan treatment, a total of 205 patients were included in the final analyses. The baseline characteristics and follow-up data at 8 weeks and 24 weeks of the 205 patients are summarized in Table 1. The mean age was 47.6 ± 12.5 years, and 105 patients (51.2%) were male. A total of 80 patients (39%) and 39 patients (19%) had hypertension and DM, respectively. Baseline median ln(uAGT/Cr) and ln(uR/Cr) were [median (interquartile range, IQR)] 3.49 (2.73–4.31) and − 1.14 (− 2.08–0.62), respectively. Baseline ln(uAGT/Cr) and ln(uR/Cr) were positively correlated with baseline uPCR (*R* = 0.455, *P* <  0.001 and *R* = 0.317, *P* <  0.001, respectively) and negatively correlated with baseline eGFR (*R* = − 0.352, *P* <  0.001 and *R* = − 0.214, *P* <  0.001, respectively).

**Table 1:** Clinical characteristics of the study population during valsartan treatment

	Baseline	8 weeks	24 weeks	***P***^**a**^	***P***^*b*^
Age (years)	47.6 ± 12.5			NA	NA
Male	105 (51.2)			NA	NA
Comorbid disease					
Hypertension	80 (39.0)			NA	NA
Diabetes mellitus	39 (19.0)			NA	NA
Mean arterial pressure (mmHg)	111.6 ± 12.0	110.9 ± 15.3	110.4 ± 12.6	0.510	0.142
Blood sample					
BUN (mg/dL)	20.1 ± 7.83	20.4 ± 8.04	21.2 ± 9.39	0.388	< 0.001
Serum creatinine (mg/dL)	1.27 ± 0.46	1.30 ± 0.48	1.32 ± 0.53	0.001	< 0.001
eGFR (mL/min/1.73m^2^)	63.2 ± 28.8	61.0 ± 27.5	61.6 ± 29.7	0.003	0.009
Serum albumin (mg/dL)	4.02 ± 0.45	4.04 ± 0.58	4.06 ± 0.58	0.193	0.301
Serum cholesterol (mg/dL)	181.8 ± 35.6	180.4 ± 36.4	178.4 ± 33.9	0.429	0.245
Serum uric acid (mg/dL)	6.91 ± 1.86	7.02 ± 1.88	6.94 ± 1.77	0.264	0.847
Urine sample					
uPCR (mg/mg)	1.85 (1.34–2.76)	1.69 (0.99–2.57)	1.40 (0.81–2.58)	< 0.001	< 0.001
ln(UAGT/Cr) (ug/g)	3.49 (2.73–4.31)	3.36 (2.21–4.06)	3.16 (2.17–4.26)	0.079	0.005
ln(UR/Cr) (pg/g)	−1.14 (−2.08–0.62)			NA	NA
uNa/Cr (mmol/mmol)	11.4 (7.2–18.8)	11.0 (6.8–17.5)	11.7 (8.1–17.3)	0.242	0.185

Abbreviations: *BUN* blood urea nitrogen, *eGFR* estimated glomerular filtration rate, *NA* not applicable, *uAGT/Cr* urinary angiotensinogen/creatinine ratio, *uNa/Cr* urinary sodium/creatinine ratio, *uPCR* urinary protein/creatinine ratio, *uR/Cr* urinary renin/creatinine ratio

Continuous variables with a normal distribution are expressed as the mean ± standard deviation, and those with non-normal distribution are expressed as the median (interquartile range). Categorical variables are expressed as number (percentage). Variables were compared using the paired *t*-test or the Wilcoxon signed-rank test as appropriate

A *P*-value < 0.025 was considered statistically significant in this table by applying Bonferroni correction method

^a^*P*: baseline vs. 8 weeks

^b^*P*: baseline vs. 24 weeks

### Changes in proteinuria, renal function, and urinary AGT excretion during valsartan treatment

Proteinuria decreased at 8 weeks and 24 weeks from the baseline after starting valsartan. However, there were no significant difference between proteinuria at 8 weeks and at 24 weeks (baseline vs. 8 weeks vs. 24 weeks; mean uPCR, mg/mg: 2.32 ± 1.43 vs. 2.06 ± 1.63 vs 2.02 ± 1.93, *P* = 0.003). The eGFR also decreased slightly at 8 weeks similar to proteinuria (baseline vs. 8 weeks vs. 24 weeks; mean eGFR, mL/min/1.73m^2^: 63.2 ± 28.8 vs. 61.0 ± 27.5 vs. 61.6 ± 29.7, *P* = 0.003). Ln(uAGT/Cr) decreased at 24 weeks after starting valsartan [baseline vs. 24 weeks; median ln(uAGT/Cr), μg/g: 3.49 (2.73–4.31) vs. 3.16 (2.17–4.26), *P* = 0.005].

### Baseline urinary RAS activity by proteinuria decrement

We compared baseline ln(uAGT/Cr), ln(uR/Cr), and Δln(uAGT/Cr) between patients with a uPCR decrement ≥1 mg/mg (decrement group) and patients with an uPCR decrement < 1 mg/mg (non-decrement group) at 24 weeks after starting valsartan (Table 2). Baseline ln(uAGT/Cr) was significantly higher in the decrement group compared to the non-decrement group [median (IQR) in the decrement vs. the non-decrement groups:3.92 (3.39–4.59) vs. 3.29 (2.53–4.21), *P* = 0.004]. Baseline ln(uR/Cr) was also significantly higher in the decrement group compared to the non-decrement group [median (IQR) in the decrement vs. the non-decrement groups: − 0.35 (− 1.96–1.73) vs. -1.25 (− 2.11–0.47), *P* = 0.047]. Ln(uAGT/Cr) decreased more in the decrement group compared to the non-decrement group at 24 weeks [median (IQR) of Δln(uAGT/Cr) in the decrement vs. the non-decrement groups: − 0.76 (− 2.07–0.80) vs. 0.00 (− 0.79–0.63), *P* <  0.001, respectively]. Doses of valsartan or baseline mean arterial pressure between the decrement and non-decrement group were similar. Baseline proteinuria levels were higher in the decrement group than the non-decrement group.

**Table 2:** Urinary AGT and renin levels and related factors according to the degree of proteinuria decrement at 24 weeks of valsartan treatment

	Non-decrement^**a**^ (***n*** = 152)	Decrement^**b**^ (***n*** = 53)	***P***
Valsartan dose at 24 weeks			0.668
320 mg	96 (63.2%)	31 (58.5%)	
160 mg	48 (31.6%)	18 (34.0%)	
80 mg	8 (5.3%)	4 (7.5%)	
Baseline uPCR	1.64 (1.22–2.49)	2.66 (1.84–3.95)	< 0.001
Baseline MAP	111.4 ± 12.0	112.1 ± 12.3	0.713
Baseline ln(uAGT/Cr) (μg/g)	3.29 (2.53–4.21)	3.92 (3.39–4.59)	0.004
Baseline ln(uR/Cr) (pg/g)	−1.25 (−2.11–0.47)	−0.35 (− 1.96–1.73)	0.047
Δln(uAGT/Cr) (μg/g)^c^	0.00 (−0.79–0.63)	− 0.76 (−2.07–0.80)	< 0.001

Abbreviations: *MAP mean arterial pressure, uAGT/Cr* urinary angiotensinogen/creatinine ratio, *uPCR* urinary protein/creatinine ratio, *uR/Cr* urinary renin/creatinine ratio

^a^Non-decrement: patients with uPCR decrement < 1 mg/mg

^b^Decrement: patients with uPCR decrement ≥1 mg/mg

^c^Δln(uAGT/Cr) = [ln(uAGT/Cr) at 24 weeks] - [baseline ln(uAGT/Cr)]

### Predictive factors for the antiproteinuric effects of valsartan

We conducted a logistic regression analysis to identify predictive factors for proteinuria decrement (decrease in uPCR ≥1 mg/mg at 24 weeks) (Table 3). The univariable analysis found DM, ln(uAGT/Cr), and ln(uR/Cr) were associated with proteinuria decrement. Subsequent multivariable analysis identified baseline ln(uAGT/Cr) as an independent predictor of proteinuria decrement (OR 1.372, 95% CI, 1.068–1.762, *P* = 0.013).

**Table 3:** Predictive factors for proteinuria decrement^a^ after 24 weeks of valsartan treatment

	Univariable			Multivariable		
	OR	95% CI	***P***	OR	95% CI	***P***
History of hypertension	1.150	0.608–2.174	0.667			
History of DM	2.105	1.005–4.411	0.049	2.091	0.964, 4.536	0.062
Previous RAS inhibitor use	0.651	0.354–1161	0.143			
MAP (mmHg)	1.007	0.981–1.003	0.600			
eGFR (mL/min/1.73m^2^)	1.006		0.256			
Baseline ln(uAGT/Cr) (μg/g)	1.387	1.082–1778	0.010	1.338	1.035, 1.729	0.026
Baseline ln(uR/Cr) (pg/g)	1.199	1.022–1.407	0.026	1.073	0.901, 1.278	0.430
ΔuNa/Cr (mmol/mmol)^b^	0.877	0.693–1.111	0.227			

Abbreviations: *CI* confidence interval, *DM* diabetes mellitus, *eGFR* estimated glomerular filtration rate, *MAP* mean arterial pressure, *OR* odds ratio, *RAS* renin angiotensin system, *uAGT/Cr* urinary angiotensinogen/creatinine ratio, *uNa/Cr* urinary sodium/creatinine ratio, *uPCR* urinary protein/creatinine ratio, *uR/Cr* urinary renin/creatinine ratio

Multivariable logistic regression analysis was conducted with variables with *P* < 0.1 in the univariable models

^a^Proteinuria decrement: decrease in uPCR ≥ 1 mg/mg

^b^ΔuNa/Cr = [uNa/Cr at 24 weeks] - [baseline uNa/Cr]

Subgroup analysis regarding long-term renal outcome was performed in 56 patients who agreed to provide the information and were followed-up for 5 years after the end of study period. None of the patients progressed to ESRD. The characteristics and urinary AGT and renin profiles of these patients were summarized supplementary Table 1. Mean arterial pressure at 5 years was not different between the patients with uPCR < 1 mg/mg and 1 ≥ mg/mg at 5 years. The eGFR at 5 years was higher in patients with uPCR < 1 mg/mg compared with those with uPCR 1 ≥ mg/mg at 5 years (61.8 ± 18.9 vs 45.0 ± 21.6 mL/min/1.73 m^2^, *P* = 0.003). The Δln(uAGT/Cr) at 24 weeks was an independent predictor for uPCR < 1 mg/mg at 5 years in the models adjusted for DM, hypertension, baseline eGFR, baseline uPCR, and ΔuPCR at 24 weeks (Table 4). DM was the only predictive factor for a change in eGFR at 5 years (eGFR at 5 years minus baseline eGFR) (β − 15.038, *P* <  0.001) (supplementary Table 2).

**Table 4:** Predictive factors for uPCR < 1 mg/mg at 5 years posttreatment

	Model 1^**a**^			Model 2^**b**^			Model 3^**c**^		
	OR	95% CI	***P***	OR	95% CI	P	OR	95% CI	***P***
ln(uAGT/Cr) (μg/g)	0.971	0.654–1.441	0.882	1.548	0.868–2.760	0.139	0.898	0.586–1.374	0.620
Δln(uAGT/Cr) (μg/g) ^d^	0.361	0.195–0.668	0.001	0.287	0.126–0.654	0.003	0.379	0.201–0.715	0.003
ln(uR/Cr) (pg/g)	0.945	0.652–1.363	0.753	1.105	0.733–1.666	0.633	1.020	0.691–1.505	0.922

Abbreviations: *CI* confidence interval, *OR* odds ratio, *uAGT/Cr* urinary angiotensinogen/creatinine ratio, *uR/Cr* urinary renin/creatinine ratio

^a^Model 1: Adjusted for diabetes mellitus, hypertension, and baseline eGFR

^b^Model 2: Adjusted for baseline uPCR, diabetes mellitus, hypertension, and baseline eGFR

^c^Model 3: Adjusted for ΔuPCR at 24 weeks (uPCR at 24 weeks - baseline uPCR), diabetes mellitus, hypertension, and baseline eGFR

^d^Δln(uAGT/Cr) = [ln(uAGT/Cr) at 24 weeks] - [baseline ln(uAGT/Cr)]

## Discussion

This study demonstrates that baseline urinary AGT excretion and changes in urinary AGT levels by ARBs have prognostic potential in predicting the antiproteinuric effects of ARBs in patients with overt proteinuria. Patients with higher baseline urinary AGT excretion showed significant antiproteinuric effects of ARBs. In addition, overt proteinuria disappeared during the 5 years of follow-up in patients with a significant decrease in urinary AGT after short-term (24 weeks) valsartan treatment. These long-term effects were independent of a decrease in proteinuria during the short-term valsartan treatment.

The antiproteinuric effects of ARBs were associated with baseline urinary AGT and urinary renin levels in our study. In our previous study including biopsy-proven glomerulonephritis patients, patients with high urinary AGT and renin showed significantly decreased proteinuria and increased eGFR during RAS-inhibitor treatment [8]. However, another study of patients with non-diabetic kidney disease with substantial proteinuria reported that the percent change in urinary AGT, not baseline urinary AGT, was associated with the percentage change in proteinuria during losartan treatment [16]. These conflicting results from previous studies may be caused from differences in underlying kidney disease, the small number of patients, and the short follow-up period. In patients with the same extent of proteinuria, the intrarenal RAS activity and the response to RAS-inhibitors may vary depending on the type of kidney disease. This study included a relatively large number of patients with diverse proteinuric kidney diseases, including diabetic or hypertensive nephropathy and glomerulonephritis, and approximately 30% of patients were followed up for 5 years. Therefore, we believe that our results reflect overall clinical relevance of urinary AGT excretion during treatment with RAS inhibitors for overt proteinuria.

Consistent with previous reports, urinary AGT decreased during valsartan treatment in our study. Although one animal study reported that the primary source of renal angiotensin II is liver-derived AGT [23], other studies have demonstrated that AGT is mainly produced in the proximal tubules and secreted into the tubular lumen [24, 25]. Plasma AGT appears to be unfiltered in the glomeruli due to its molecular weight (52–64 kDa) and negative charge [26]. Urinary AGT excretion reflects intrarenal RAS activity rather than filtration of plasma AGT in humans, but those studies only included patients with minimal proteinuria [9, 27, 28]. Like albumin, if the integrity of the filtration barrier is damaged, a large amount of plasma AGT can be filtered and excreted into the urine [8, 23]. Therefore, we cannot exclude the possibility that some portion of urinary AGT in patients with heavy proteinuria might be filtered from systemic AGT through a damaged filtration barrier. However, decreased urinary AGT excretion at 24 weeks was associated with antiproteinuric effects of valsartan up to 5 years later, independent of a decrease in proteinuria at 24 weeks, supporting the value of urinary AGT as a biomarker reflecting intrarenal RAS activity.

There was no correlation between urinary AGT or renin excretion and the change in eGFR at 24 weeks as well as at 5 years, although baseline urinary AGT and renin excretion were negatively correlated with baseline eGFR. Urinary AGT and renin excretion are negatively correlated with eGFR [21, 27, 29], but the relationship between urinary AGT or renin excretion and changes in renal function has not been well studied. Previous studies have suggested that baseline AGT excretion may have limited value as a prognostic factor in predicting changes in renal function during RAS-inhibitor treatment, although it has a negative correlation with changes in renal function without RAS inhibitors [8, 27, 30, 31]. RAS inhibitors seem to attenuate the differences in renal function changes between patients with high and low urinary AGT by inhibiting intrarenal RAS and subsequently decreasing proteinuria. However, a large-scale study with a long-term follow-up period is needed to clarify this issue.

Previous studies have suggested that urinary renin may be a better marker of intrarenal RAS activity than urinary AGT, especially in patients with heavy proteinuria [18]. However, our study showed that urinary renin excretion was not superior to urinary AGT for predicting the antiproteinuric effects of ARBs. The values of urinary renin excretion were relatively lower and showed a more skewed distribution compared to urinary AGT even though a previously verified method for measuring urinary renin was used [8]. Since a significant number of patients showed very low urinary renin levels close to the detection limit at the baseline, we did not measure urinary renin at 24 weeks. Urinary AGT seems to be a better biomarker for evaluating intrarenal RAS activity based on the current methodological limitations of urinary renin measurement.

There were several limitations in this study. First, our study included heterogeneous kidney diseases because overt proteinuria was the main inclusion criterion. The response to RAS inhibitors can be affected by the underlying kidney disease, and further studies enrolling a more homogenous population are required. However, our study has clinical relevance as it shows the overall relevance of urinary AGT or renin in patients with overt proteinuria receiving ARB treatment. Second, a substantial number of patients were excluded due to insufficient data and a relatively small portion of patients was followed up for 5 years. Although these issues might cause selection bias, our study has a relatively large number of patients with overt proteinuria and the longest follow-up period compared with many previous studies. Third, the methodological limitation in measuring urinary renin activity also hindered the analysis although we used a previously verified method. The lack of serial follow-up data of urinary renin at 8 and 24 weeks after starting valsartan may limit generalization of our study results. Further studies using a more sensitive and precise method in a larger number of patients are required to investigate the clinical predictive usefulness of urinary renin levels in patients with overt proteinuria.

## Conclusions

Our study demonstrated the clinical relevance of urinary AGT as a biomarker for predicting antiproteinuric effects of RAS inhibitors in both short-term and long-term follow-up periods. Both baseline urinary AGT excretion and urinary AGT changes during a short-term follow-up period may be used as potential surrogate markers for the effectiveness of RAS inhibitor treatment for patients with overt proteinuria.

## Supplementary information

**Additional file 1: Supplementary table 1.** Characteristics and urinary AGT and renin of 56 patients who were followed up to 5 years. **Supplementary table 2.** Predictive factors for change in GFR^a^ from baseline to 5 years in 56 patients.

## Data Availability

The datasets used and/or analyses during the current study are available from the corresponding author on reasonable request. The requirement should first be submitted to ethical committee.

## Abbreviations

AGT: Angiotensinogen
ARB: Angiotensin receptor blocker
CKD: Chronic kidney disease
uAGT/Cr: Urinary angiotensinogen/creatinine ratio
eGFR: Estimated glomerular filtration rate
RAS: Renin angiotensin system
uNa/Cr: Urinary sodium/creatinine ratio
uPCR: Urinary protein/creatinine ratio
uR/Cr: Urinary renin/creatinine ratio
