# Supplementary material for: Urinary angiotensinogen as a surrogate marker predicting the antiproteinuric effects of angiotensin receptor blockers in patients with overt proteinuria: a multicenter prospective study
Source: BMC Nephrol. 2020 May 1;21:180. doi: 10.1186/s12882-020-01825-6 (PMC7227290; doi:10.1186/s12882-020-01825-6)
Supplement: Supplementary file 1 — Additional file 1: Supplementary table 1. Characteristics and urinary AGT and renin of 56 patients who were followed up to 5 years. Supplementary table 2. Predictive factors for change in GFRa from baseline to 5 years in 56 patients. [file 12882_2020_1825_MOESM1_ESM.docx]

**Supplementary table 1.** Characteristics and urinary AGT and renin of 56 patients who were followed up to 5 years

|  | **Total**  **(n =56)** | **uPCR < 1 g/g**  **at 5 years**  **(n = 27)** | **uPCR > 1 g/g**  **at 5 years**  **(n = 29)** | ***P*** |
| --- | --- | --- | --- | --- |
| uPCR (g/g) at 5 years | 1.08 (0.53, 2.56) | 0.50 (0.20, 0.78) | 2.30 (1.50, 3.65) | < 0.001 |
| eGFR (mL/min/ 1.73m^2^) at 5 years | 53.1 ± 21.8 | 61.8 ± 18.9 | 45.0 ± 21.6 | 0.03 |
| MAP (mmHg) at 5 years | 91.0 ± 11.0 | 92.2 ± 10.3 | 89.5 ± 11.9 | 0.472 |
| Baseline ln(uAGT/Cr) (μg/g) | 3.52 (2.03, 4.33) | 3.26 (1.84, 3.93) | 3.78 (2.80, 4.56) | 0.197 |
| Baseline ln(uR/Cr) (pg/g) | 0.32 (-1.23, 2.49) | -0.02 (-1.49, 1.16) | 0.60 (-0.76, 2.60) | 0.197 |
| Δln(uAGT/Cr) (μg/g)^a^ at 24 wks | -0.50 (-1.73, 0.017) | -1.51 (-2.75, -0.42) | -0.08 (-0.82, 0.48) | < 0.001 |

*Abbreviations: eGFR estimated glomerular filtration rate, MAP mean arterial pressure, uAGT/Cr* urinary angiotensinogen/creatinine ratio, *uPCR* urinary protein/creatinine ratio*, uR/Cr* urinary renin/creatinine ratio

^a^Δln(uAGT/Cr) = [ln(uAGT/Cr) at 24 weeks] - [baseline ln(uAGT/Cr)]

**Supplementary Table 2.** Predictive factors for change in GFR^a^ from baseline to 5 years in 56 patients

|  | **Univariable** | |
| --- | --- | --- |
|  | **β coefficient** | ***P*** |
| History of hypertension | -3.769 | 0.321 |
| History of diabetes mellitus | -15.038 | < 0.001 |
| Baseline ln(uAGT/Cr [ug/g]) | -0.696 | 0.577 |
| Baseline ln(uR/Cr [pg/g]) | -0.731 | 0.498 |
| Δln(uAGT/Cr [ug/g])^b^ | -0.809 | 0.525 |
| Baseline uPCR (mg/mg) | -2.023 | 0.175 |

Multivariable analysis was not performed because there was no variable with P value < 0.1 in univariable regression models.

^a^Change in GFR = [eGFR at 5 years] – [baseline eGFR]

^b^Δln(uAGT/Cr) = [ln(uAGT/Cr) at 24-weeks] – [baseline ln(uAGT/Cr)]

eGFR, estimated glomerular filtration rate; uAGT/Cr, urinary angiotensinogen/creatinine ratio; uPCR, urinary protein/creatinine ratio, uR/Cr, urinary renin/creatinine ratio
